# General practitioners’ perceptions of working with the certification of sickness absences following changes in the Swedish social security system: a qualitative focus-group study

**DOI:** 10.1186/s12875-015-0238-5

**Published:** 2015-02-21

**Authors:** Lars Carlsson, Linda Lännerström, Thorne Wallman, Inger K Holmström

**Affiliations:** Department of Public Health and Caring Sciences, Family Medicine and Preventive Medicine Section, Uppsala University, Box 564, 75122 Uppsala, Sweden; Centre for Clinical Research Dalarna, Uppsala University, Falun, Sweden; Centre for Clinical Research Sörmland, Uppsala University, Eskilstuna, Sweden; School of Health, Care and Social Welfare, Mälardalen University, Västerås, Sweden; Department of Public Health and Caring Sciences, Health Services Research Section, Uppsala University, Uppsala, Sweden

**Keywords:** General practitioners, Primary health care, Focus group discussions, Sick leave, Certification of sickness absence, Social security system

## Abstract

**Background:**

Many physicians in Sweden, as well as in other countries, find the matter of certification of sickness absence (COSA) particularly burdensome. The issuing of COSAs has also been perceived as a work-environment problem among physicians. Among general practitioners (GPs) are the highest proportion of physicians in Sweden who experience difficulties with COSA. Swedish authorities have created several initiatives, by changing the social security system, to improve the rehabilitation of people who are ill and decrease the number of days of sick leave used. The aim of this study was to describe how GPs in Sweden perceive their work with COSA after these changes.

**Methods:**

A descriptive design with a qualitative, inductive focus-group discussion (FGD) approach was used.

**Results:**

Four categories emerged from the analysis of FGDs with GPs in Sweden: *1) Physicians’ difficulties in their professional role; 2) Collaboration with other professionals facilitates the* COSA*; 3) Physicians’ approach in relation to the patient; 4) An easier* COSA *process.*

**Conclusions:**

Swedish GPs still perceived COSA to be a burdensome task. However, system changes in recent years have facilitated work related to COSA. Cooperation with other professionals on COSA was perceived positively.

## Background

Issues with extended sickness absence have gained increased attention in recent years, especially in northwestern Europe. In Sweden, there has been intense debate on sickness absence issues over the past decade because of the rapid and large increase in the number of sickness absence days granted by. From 1998 to 2003, the total number of sickness absence days more than doubled. Thereafter, the number of days decreased to a level even lower than it had been in 1998, and started increasing again in 2010 [1].

Swedish authorities have introduced several initiatives to improve the rehabilitation of people who are ill and reduce absences due to sickness. Some are economic incentives for the county councils, while others are changes in rules and regulations. Such incentives include the “Sick Leave Billion” (Sjukskrivningsmiljarden), with the government spending SEK 1 billion each year from 2006 to 2013 to provide Sweden’s 21 county councils with financial incentives to continue their efforts to enhance the quality and efficiency of the sickness certification process [2].

Another incentive is the “Rehabilitation Guarantee” (Rehabiliteringsgarantin), whereby the authorities have paid the county councils SEK 1 billion each year from 2009 to 2013. This is a national programme providing cognitive behavioural therapy to patients with light or moderate mental and behavioural disorders, and multimodal rehabilitation for those with musculoskeletal-related pain in the back, neck and shoulders. The programme was introduced in 2008, with the purpose of preventing sickness absence and increasing the rate of return to work among patients with these diagnoses [3]. The county councils have been encouraged through economic incentives from the “Rehabilitation Guarantee” to set up rehabilitation teams with a coordinator at primary health care centres. Most often, the rehabilitation coordinator has been a physiotherapist, psychotherapist or occupational therapist. Some rehabilitation coordinators have taken on a more prominent role by leading the rehabilitation work, whereas elsewhere, the task is left to the treating physician. The teams usually consist of all four professions and are co-located at the primary health care centre.

An amendment to the 2008 legislation called the “Rehabilitation Chain” (Rehabiliteringskedjan) dictates that work ability should be assessed in relation to the patient’s regular work tasks within the first 90 days of sick leave. For Days 91–180, work ability is assessed in comparison with other work tasks at the patient’s workplace. From Day 181, work ability is assessed in comparison to other normally occurring tasks within the entire labour market [4].

As an aid to physicians, in 2007, “Guidelines for Sick Leave” (Försäkringsmedicinskt beslutsstöd) were introduced by the Swedish National Board of Health and Welfare, offering advice regarding reasonable sick-leave time for specific diagnoses [5]. The aim was to make COSA consistent and coherent, and to achieve a legally secure COSA process.

The Swedish Social Insurance Agency has, in recent years, also become stricter in its assessment of the sickness absence note. To be approved, the sick note must be completed fully in accordance with the “DFA Chain” (DFA-kedjan), which means that diagnosis, functional impairment and activity limitation should be logically linked for the sickness absenteeism to be approved [6].

COSA issues are common not only in family medicine, but also in orthopaedics, rehabilitation, oncology, occupational health and psychiatry [7]. Several reports state that COSA issues are perceived as problematic among physicians [7,8]. GPs in Sweden, as well as in other countries, experience extensive problems associated with COSA assignment, according to several studies [9-16]. Norwegian GPs, for example, consider issuing COSA for patients with composite health complaints to be challenging and burdensome [17]. Furthermore, new COSA standards focusing on functional assessment caused conceptual and practical problems among GPs in Norway [18]. Physicians at primary health care centres in Sweden experience barriers to good COSA practices, both within the health care system and outside among other stakeholders who are involved in the rehabilitation of patients [19]. Among GPs are the highest proportion of physicians in Sweden who experience difficulties with COSA [7,8,20]. Issuing COSA has also been perceived as a work-environment problem among physicians in Sweden and other countries [8,12,21-25].

Hence, a large proportion of physicians find COSA particularly burdensome. Many changes have been made in Swedish COSA regulation and practice in recent years. The aim of this study was to describe how GPs in Sweden perceive their work with COSA following these changes.

## Methods

### Design

A descriptive design with a qualitative, inductive focus-group discussion (FGD) approach was chosen.

### Sample and setting

Participants were selected strategically from different parts of Sweden using our professional network of GPs, with the goal of obtaining wide variation. The informants were enrolled from rural areas and cities with different population sizes and with varying professional experience and gender. The goal was to achieve *"*maximum variation in sampling*"* [26]. The informants received verbal and written information before being asked to sign the informed consent and answer written demographic questions about age, gender, work experience and their own sick-leave experiences.

A total of five FGDs comprising 22 GPs were conducted (Table 1). All FGDs were conducted in Swedish, and the analysis was also conducted in Swedish by the authors, who are native speakers of Swedish. After analysis, the quotes were translated into English by the authors and checked by a native English-speaking translator.

**Table 1:** **Participants in the focus group discussions**

**Person**	**Specialist in family medicine**	**Working years**	**Extent of duty**	**Personal experience of sick leave more than 7 days**	**Specially engaged in sickness absence issues**
1	Yes	28	70	Yes	0
2	Yes	7	100	0	0
3	0	1	100	0	0
4	Yes	28	100	Yes	Yes
5	Yes	12	80	Yes	0
6	Yes	1	100	0	0
7	0	-	75	0	0
8	Yes	16	100	0	Yes
9	Yes	16	100	0	0
10	Yes	18	100	0	0
11	Yes	7	65	0	0
12	Yes	32	85	0	0
13	Yes	1	100	0	0
14	Yes	4	100	0	Yes
15	Yes	6	75	0	0
16	0	6	80	Yes	0
17	0	2	100	0	0
18	0	2	80	0	0
19	0	2	100	0	0
20	0	2	100	Yes	0
21	0	1	80	0	0
22	0	1	75	Yes	0

The data gathered from the FGDs were considered to be sufficient to achieve saturation when similar descriptions of the experience of COSA assignment recurred in different FGDs.

The focus groups had three to seven participants each, and a total of ten participants (45%) were women. Their ages varied between 30 and 63 years, with an average age of 45 years. The informants’ experience of working as a GP ranged from 1 to 32 years, with an average tenure of 9 years. Twelve GPs worked full-time, and the others worked part-time. On average, they worked 76.5% of a full-time load. Six of the GPs had taken sick leave for more than 7 days. Three were especially engaged in sickness absence issues. The majority of the GPs were public employees, but one FGD consisted of four private GPs. One group consisted of physicians in training to become specialists in family medicine; the rest of the groups consisted of specialists. The FGDs were conducted at the physicians’ workplace. The informants in each FGD knew each other, as they worked in the same location.

### Data collection

FGDs are an effective way to obtain as wide a spectrum as possible of views on a research question [27]. FGDs are also particularly effective in capturing variation in the opinions of a group [28]. In FGDs, people are encouraged to talk to one another, ask questions, exchange anecdotes and comment on each other’s experiences and points of view [29]. In an FGD, one can more easily discuss sensitive and taboo topics through group interaction [27]. The goal of an FGD is not to reach consensus or find solutions, but rather, to highlight different views on an issue [27]. According to Morgan, the FGD is suitable when there are considerable differences between people’s perceptions and when you wish to understand the differences [30]. The present FGDs were semi-structured, with open questions. A discussion guide was constructed based on previous studies and clinical experience, to ensure that important areas were covered (see “Discussion guide”). The informants were encouraged to talk freely about their experiences related to COSA assignment. The FGDs were conducted in late 2011 and 2012. The first author played the role of moderator in the discussions, and the second author was an observer. Each FGD lasted 50–90 minutes. The discussions were recorded with a digital voice recorder and were then transcribed verbatim. The study was approved by the Regional Ethical Review Board at Uppsala University (Dnr 2011/466).

### Discussion guide

- How do you view your work with sick leave?
- Do you feel it is common for doctor and patient to have different opinions about the need for sick leave?
- How do you handle this?
- How was the last sick-listing you remember?
- Why are you thinking of it?
- What was your most difficult sickness certification?
- Why this one in particular?
- How do you experience assessing work ability?
- Could sick leave notes be a health and safety problem?
- How do you believe the sick-listing process been affected by the changes in the social security system?

### Data analysis

Qualitative analysis was performed using conventional manifest content analysis [31,32]. As there was a lack of predetermined theory, we used an inductive approach to analyse the data [33]. In qualitative manifest content analysis, meaning units are classified in subcategories after coding. After continued abstraction and analysis of the subcategories, they are grouped into categories. After listening to the recorded interviews and checking the verbatim transcribed recordings, an initial identification of meaning units was done by the first author. Thereafter, this initial coding was scrutinized and revised by the last author, who is an expert in qualitative methods. The findings were thereafter discussed by the whole group of authors, until consensus was reached. A total of 349 meaning units were identified from the five FGDs. All meaning units were included in the analysis. Further abstraction and content analysis, according to Graneheim and Lundman, including coding, resulted in 23 subcategories that could be grouped into four categories. Analysis was completed by all the authors together (Table 2) [31].

**Table 2:** **Analysis structure**

**Meaning unit**	**Code**	**Subcategory**	**Category**
When you’re the treating physician, so to say, you have a role. When you're issuing a sick note as a physician then that’s a completely different role as well… You're the Social Insurance Agency’s extended arm or a public authority person, which has nothing to do with the role of treating physician.	Two roles	Dual roles	**Physicians’ difficulties in their professional role**
But there I think we've learned to take more help from other professionals who at our medical centres. So I feel it’s improved a lot, that we’ve begun to understand that we’re not the only ones who have to manage this.	Cooperation with other professionals	Cooperation	**Collaboration with other professionals facilitates the COSA**
But there are actually few really justified sick leaves; you can question a lot of the sick leaves.	Few justified sick leaves	Doubts	**Physicians’ approach in relation to the patient**
The pressures to be placed on sick leave have gotten much lower; today people won’t come and demand to be placed on sick leave.	Decreased claim	Less demand for sick leave	**An easier COSA process.**

## Results

Four categories emerged from the analysis of the FGDs with GPs in Sweden discussing how they perceived COSA assignment. Each category will be discussed in greater detail below.

1. *Physicians’ difficulties in their professional role*
2. *Collaboration with other professionals facilitates the COSA*
3. *Physicians’ approach in relation to the patient*
4. *An easier COSA process*

### Physicians’ difficulties in their professional role

The physicians found it burdensome to have dual roles as the patient’s physician and as an official issuing sick notes to the Social Insurance Agency. These dual roles were not only exhausting to the physicians, but were most likely also confusing for patients: *“When you’re the treating physician, so to say, you have a role. When you're issuing a sick note as a physician, then that’s a completely different role as well… You're the Social Insurance Agency’s extended arm or a public authority person, which has nothing to do with the role of treating physician”* (FGD C).

The assessment of the impact of the patient’s symptoms on work ability was experienced as being difficult. The GPs at the health centres had very little knowledge about the patient’s actual situation at the workplace, and had to rely entirely on the patient’s description of working conditions: “*We should always consider what tasks the patient can or cannot cope with, but really we have no idea what that workplace looks like*” (FGD D).

The GPs felt doubts concerning whether sickness absence always helps the patient. There was concern that it could be a way of escaping from workplace problems instead of solving them, thereby paradoxically slowing down clinical recovery. *“In a sense, you could say that it’s the technical part, the really tricky part, it’s to determine whether… whether it’s good or bad for the patient to sick-list him”* (FGD B).

The GPs reported that the COSA decision was complicated by the physician’s empathy for the patient, subjective judgments and uncertainties about working conditions. *“Thus, our sympathies, we have…our empathy for the patient…compassion that we have. It’s not the same as their being truly eligible for sickness absence*” (FGD C).

COSA patients were perceived to be very burdensome compared with other patient categories that one might expect to be more burdensome. “*There were people who died, and there was cancer diagnosis, and there was sudden infant death. This was the way it was. But that wasn’t what brought you to your knees. Instead, what made me half broken-down occasionally, that was sick-leave cases”* (FGD E)*.* This could possibly be explained by the aforementioned difficulties with the physicians being torn by dual roles, their lack of knowledge of the workplace, their uncertainty whether sickness absence would really help the patient, and their empathy for the patient.

### Collaboration with other professionals facilitates the COSA

The physicians said that they perceived collaboration with other professionals as being helpful in the management of COSA patients: “*But there I think we've learned to take more help from other professionals at our medical centres. So I feel it’s improved a lot, that we’ve begun to understand that we’re not the only ones who have to manage this”* (FGD E); “*We have very good help at this health centre from the rehabilitation coordinator. Thus, it’s a huge asset for patients and a very big relief for us doctors”* (FGD E)*.* We interpret this as, the development of cooperation with other professionals like physiotherapists, psychotherapists and occupational therapists at primary health care centres in Sweden has been positive for physicians and patients. The cooperation may have been stimulated by the “Rehabilitation Guarantee”.

### Physicians’ approach in relation to the patient

Informants reported, “*The willingness to work; we seldom talk about it, but it’s statistically normally distributed like most other things”* (FGD A) and *“But there are actually few really justified sick leaves; you can question a lot of the sick leaves”* (FGD C). These perceptions could be interpreted as the physicians experiencing ambivalence or conflicting emotions during patient contact for COSA. Doubts and mistrust sometimes affected how they handled COSA.

Clear frustration also emerged among the informants when handling COSA issues: *“Everyone knows. The Social Insurance Agency knows, the Employment Agency knows, everybody knows he’ll never work again in his entire life, but it’s not because he can’t work but because he hasn’t intended to do it, and then you can’t get…you can’t get someone to work if he doesn’t want to work”* (FGD E).

The physicians reported that patients with problems in rehabilitation and who show slow improvement create feelings of stress: *“You feel this stress yourself, when you don’t get the patient well enough to start working again quickly enough, you feel…it feels a little bit…yeah, you…that you…like a failure, partly”* (FGD D)*.* We interpreted this as feelings of frustration and stress that had a strong negative effect on the experience of sickness absence assignment for physicians at primary health care centres in Sweden.

The informants perceived focussing on patients’ problems to be problematic, thereby emphasizing the problems, rather than the patients’ strengths to overcome weaknesses. *“Consolidating the weaknesses by highlighting them. The goal should always be to emphasize strengths to overcome the weaknesses”* (FGD E)*.* We believe doubts, frustration, perceived stress and uncertainty of how to manage the patient’s problem in the best possible way also contributed to physicians’ difficulties in their professional role. How physicians communicated with the patients about their views of the problem, and where the line was drawn, was highly individual.

### An easier COSA process

The informants stated: “*The pressures to be placed on sick leave have gotten much lower; today people won’t come and demand to be placed on sick leave”* (FGD B). This could be interpreted to mean that, due to decreased claims and reduced expectations for sick leave from patients, the COSA process has become easier.

The informants stated that: “*No, it’s the case that you can blame the Social Insurance Agency; I’m not the one who says you have to work – it's the rules that say that”* (FGD A). This could be interpreted to mean that physicians sometimes deferred the responsibility and decisions to the Swedish Social Insurance Agency. Formally, the Swedish Social Insurance Agency also previously decided whether sick leave would be approved or not. With the new rules, their responsibility in this role became evident. The “Guidelines for Sick Leave”, issued in 2007, offer advice regarding reasonable sick leave time for specific diagnoses. The “Rehabilitation Chain” from 2008 stipulates time limits for which work tasks working capacity should be assessed against.

**Figure 1:**
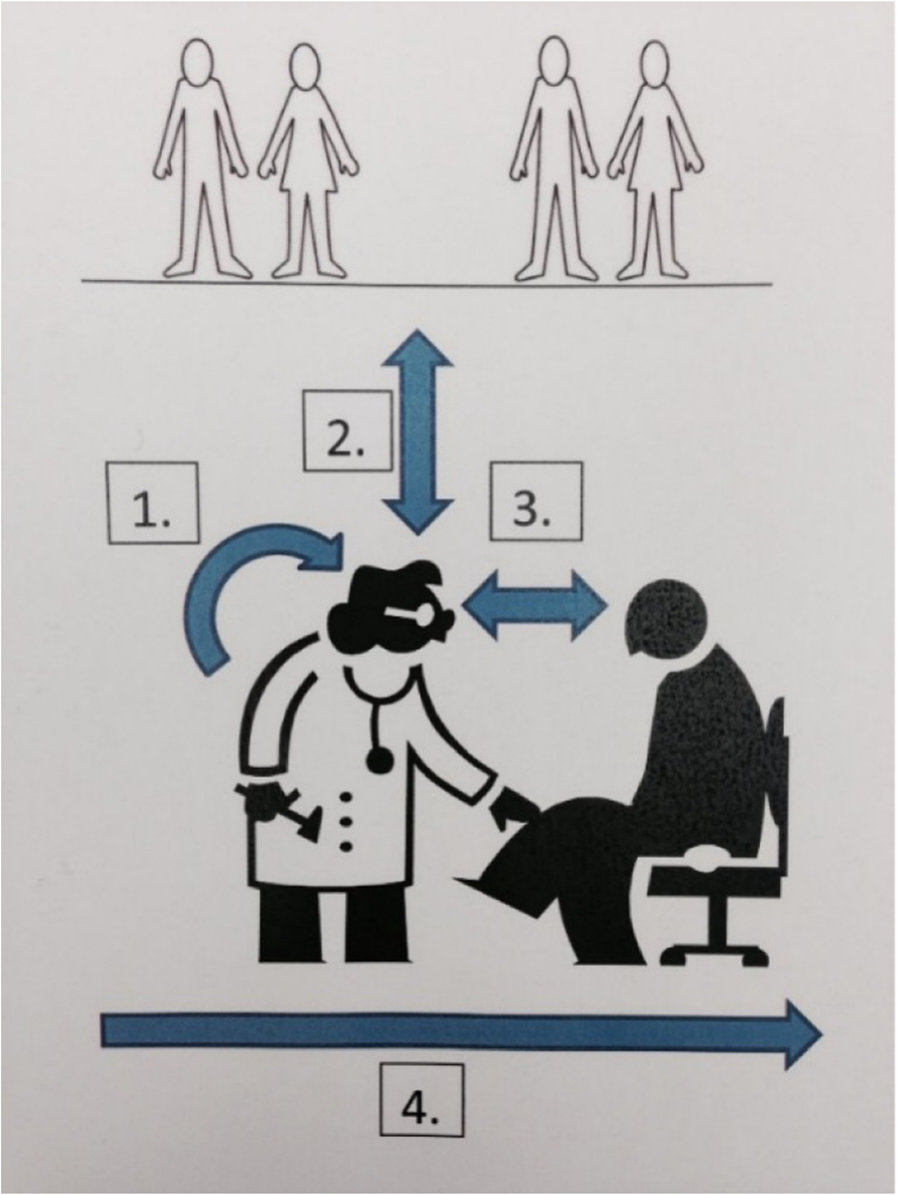
**Illustration of the categories that emerged from the FGDs.** 1. Physicians’ difficulties in their professional role. 2. Collaboration with other professionals facilitates the COSA. 3. Physicians’ approach in relation to the patient. 4. An easier COSA process.

The informants stated: *“But those new rules are an advantage, I think; because previously, it was just that the patient could come and put pressure on me – ‘Now you have to put me on the sick list’ and I had, like, no choice. Now it's like this, I can say we can try, that I’ll gladly issue a sick note but we’ll leave the assessment to someone else”* (FGD E). This could be interpreted to mean that physicians perceived the new rules to be beneficial to their experience of COSA assignment. We also think collaboration with other professionals is a major contribution to a less burdensome COSA process for physicians in Swedish primary health care centres (Figure 1).

## Discussion

The most striking finding is that Swedish GPs still perceived COSA assignment to be a burdensome task. System changes that occurred in recent years aimed to improve and accelerate the rehabilitation of patients on sick leave and also facilitated work with COSA for physicians. Cooperation with other professionals on COSA cases was perceived positively by physicians at primary health care centres in Sweden.

These findings confirm previous studies’ findings that COSA may be difficult and burdensome for GPs [7,8,10-14,20]. Sometimes, COSA may also be perceived as a work-environment problem by GPs [8,12,21-24]. Additionally, persons on long-term sickness absence experienced the process of being on sick leave as very negative [34].

In this study, the problems with COSA could be attributed to GPs’ difficulties in their professional role and their approach in relating to the patient. However, the study also provides a somewhat different picture – that since the recent years’ reforms in Sweden, COSA assignment has actually become less burdensome. This is because patients are generally more aware now that they do not have a right to be on sick leave, but also because GPs are now more likely to cooperate with other professionals in handling COSA cases.

Sometimes, the physician also defers responsibility to the Swedish Social Insurance Agency by emphasizing that it is actually the Agency that determines sick-leave approval based on what is stated on the sick note.

Results with similar findings, namely, that COSA assignment has actually become less burdensome, have also emerged in quantitative studies [7]. General attitudes to sickness absence, labour market, social factors and cooperation with other stakeholders outside health care are examples of factors outside the health care system that may affect the GPs’ perception of COSA as a problematic area [19].

The “Rehabilitation Guarantee” is an initiative that aims to improve rehabilitation during sickness absence through collaboration between other professionals, like physiotherapists, psychotherapists and occupational therapists. Patients report experiencing positive effects at the symptom level, thanks to this multidisciplinary intervention [35]. Also, GPs appreciate the cooperation with other professionals in sick absence issues. In contrast, however, the number of granted sick leave days is increasing [35].

Today, multidisciplinary cooperation in sickness absence issues is common in Swedish primary health care between physicians, physiotherapists, psychotherapists and occupational therapists. Cooperation is encouraged by medical recommendations from SBU (Swedish Council on Health Technology Assessment) and financial incentives from the “Rehabilitation Guarantee”. Studies are underway to examine whether nurses may also play a larger role in providing care for patients on sick leave.

How GPs in Sweden perceive their COSA work may also be affected by other factors. Education may be a way to facilitate the handling of COSA for GPs. In two surveys of physicians in Sweden, a high proportion (up to 91%) expressed a need to develop their knowledge in insurance medicine [7,36]. The “Sick Leave Billion”, which aims to provide Sweden’s 21 county councils with financial incentives to continue their efforts to enhance the quality and efficiency of the COSA process, includes education for physicians [2]. Balint group discussions may build better doctor–patient relationships and foster greater work-related satisfaction for GPs, which in turn may facilitate COSA assignments [37]. The Swedish Social Insurance Agency administers annual surveys of the percentage of approved medical certificates, in accordance with specific criteria, which affects the county councils’ compensation from the “Sick Leave Billion” [2].

As the current study is small-scale and qualitative, the findings cannot be generalized. Transferability was pursued by including GPs from private and public health care; geographic spread including smaller towns and bigger cities in Sweden. Variations in age, gender and professional experience reflected the demographics of GPs at other primary health care centres in Sweden. Similar perceptions of COSA assignment emerged from the various FGDs. These similarities provides transferability and dependability We pursued confirmability using open questions during the FGDs and encouraged informants to talk freely about their experiences of COSA assignment. This gives credibility to the results and enhances trustworthiness of this study, according to Lincoln and Guba [38]. Because social security systems differ between countries, transferability may be reduced, although physicians and patients are comparable between countries. The strengths of this study are the rich material collected from the FGDs and the use of researcher triangulation with different competencies, i.e., two clinically active GPs and two nurses with considerable experience working in health care centres, as well as in qualitative research. The experiences and expertise of the researchers provide both emic (insider approach) and etic (a more neutral-outsider approach) perspectives on COSA practice.

Currently, there is a lack of studies on how cooperation between physicians and other professionals can best improve rehabilitation for patients, as well as working conditions for physicians and other professionals.

## Conclusions

Swedish GPs still perceive COSA assignment to be a burdensome task. System changes in recent years have, however, facilitated their work with COSA. Cooperation with other professionals on COSA patients was perceived positively by GPs.

With the aim of offering the patient the best possible help, utilizing different professionals and health care resources most effectively and promoting a better work environment, there is a need for future studies to explore which patients benefit most from an intervention, at what point during the sick-leave period the intervention should be initiated, and what content the intervention should have.

## Abbreviations

COSA: Certification of sickness absence
GP: General practitioner
FGD: Focus-group discussion
